# Primary malignant melanoma of the lower oesophagus presenting with dysphagia and upper gastrointestinal bleeding

**DOI:** 10.1186/1757-1626-1-28

**Published:** 2008-07-11

**Authors:** Quor Meng Leong, Juinn Huar Kam

**Affiliations:** 1 Department of General Surgery, Tan Tock Seng Hospital, 11 Jalan Tan Tock Seng, 308433, Singapore; 2 National Cancer Centre Singapore,11 Hospital Drive, 169610, Singapore

## Abstract

Primary malignant melanoma of the oesophagus is an exceedingly rare oesophageal neoplasm. It accounts for less than 0.1% of all primary oesophageal tumours. Less than 262 cases have been documented till today. Surgical resection with re-establishment of gastrointestinal continuity is the treatment of choice. However, haematogenic and lymphogenic metastases are common and despite advances in surgery, radiotherapy and chemotherapy, this tumour continues to be a highly aggressive neoplasm with poor prognosis. The case of a 64 year-old lady with this tumour is presented.

## Background

Primary malignant melanomas of the oesophagus are extremely rare oesophageal neoplasms and an uncommon presentation of the malignant melanoma. Less than 262 cases have been described in world literature and incidence figures quoted a range from 0.1 – 0.4 of all oesophageal carcinomas [1,2].

## Case Presentation

A 64 year-old Chinese female with well-controlled essential hypertension presented with a 2 month history of dysphagia and retrosternal discomfort and occasional blood-tinged vomitus. This was not associated with significant anorexia or weight loss. Digital rectal examination revealed malaena, the rest of the physical examination was normal and did not reveal any melanomas.

An oesophago-gastro-duodenoscopy was performed, which revealed a large exophytic tumour at the gastric cardia and gastro-oesophageal junction. Histological analysis of biopsy specimens from the tumour suggested the possibility of a malignant melanoma.

A computed tomographic scan of the thorax and the abdomen revealed the presence of irregular thickening of the gastro-oesophageal junction which extended into the lesser curve of the stomach. Small lymph nodes were also seen at the coeliac axis on the CT scan. No pulmonary lesions were noted. A whole body Tc-99m MDP bone scan did not reveal any evidence of bone metastases. Early elective surgical resection was planned as the patient had continued to suffer from intermittent upper gastrointestinal haemorrhage.

A subtotal oesphagectomy with proximal gastric resection was performed with a feeding jejunostomy. The patient was started on jejunostomy feeds on the third post-operative day to ensure early enteral nutrition. Oral feeding was commenced by the 12^th ^post-operative day after a gastrograffin swallow confirmed the absence of any leaks. The feeding jejunostomy was removed on the 16^th ^post-operative day and the patient subsequently discharged the day after.

The margins of the resected specimen were clear of malignant tissue. A 5.5 cm diameter pigmented tumour nodule located at the gastro-oesophageal junction was diagnosed as a nodular malignant melanoma of the oesophagus which extended into the stomach. Histologically, this tumour was found to be composed of epitheloid cells with pleomorphic vesicular nuclei with prominent nucleoli and intranuclear inclusions, eosinophilic cytoplasm containing abundant melanin pigment, confirmed by immunostaining with HMB 45. Two of the eight resected peri-gastric lymph nodes contained metastatic malignant melanoma.

## Discussion

While malignant melanoma is a common skin tumour, primary growth of this tumour in other organs is exceedingly uncommon. Primary malignant melanoma occurs twice as frequently in men as in women [1] The exception is Japan, with more women being diagnosed than men. The tumour most commonly occurs in the 6^th ^and 7^th ^decade, although younger patients have been documented as well [3,4] The mean age of presentation is 60.5 years. It may be found in all areas along the oesophagus, but has been located in the lower two-thirds of the oesophagus in 86% of cases [2] No predisposing factors have been identified with certainty, but benign oesophageal melanosis is believed to be pre-malignant [5].

From the increasing number of case reports, a fairly consistent clinical presentation of such patients has emerged. Most would complain of dysphagia, non-specific retrosternal pain and weight loss [6] Occasionally, haematemesis and melaena, as in our patient, are observed. Abnormal physical findings are generally absent. The mean duration of symptoms is short, approximately 3 months.

Primary malignant melanomas of the oesophagus are grossly polypoidal and may vary greatly in size [7] Biopsy specimens are occasionally misdiagnosed as poorly differentiated carcinoma, especially when the melanoma cells contain few or no melanin granules. Immmunohistochemical staining provides a more accurate pre-treatment diagnosis by identifying melanocytic specific markers such as HMB45 antibody. The exact quantification of oesophageal tumour mass is difficult but is best judged from the combination of endoscopic, CT scan and other radiological techniques. Evaluation for metastatic disease should be carried out using multiple radionucleide or CT scans. Up to 80% of metastatic lesions are identified by the use of technetium 99-labelled melanoma monoclonal antigens [4] Unfortunately, 40–49% of patients will have metastases at the time of diagnosis. The commonest sites are the paraoesophageal lymph nodes, supraclavicular lymph nodes, coeliac lymph nodes liver, lungs, and bones [1].

Surgical resection in the form of a subtotal oesophagectomy is the treatment of choice, with a mean survival of 14.8 months and a 5-year survival rate of 4%. [1,8] Palliative limited local resection may be required to maintain a swallowing capability and to enhance the patient's quality of life but has a mean survival of only 9 months. Other therapeutic options such as chemotherapy, radiotherapy, and immunotherapy provide limited benefits, even when used in conjunction with surgery [9].

## Conclusion

Primary malignant melanoma of the oesophagus is a rare but aggressive disease, often associated with metastatic spread at the time of presentation. Early detection, establishing a definitive diagnosis and effective treatment remain a challenge. Radical surgical resection has achieved the longest survival and the best prospect of a cure. Chemotherapy and radiotherapy are mainly deployed for palliative purposes only.

## Consent

Written informed consent was obtained from the patient for publication of this case report. A copy of the written consent is available for review by the Editor-in-Chief of this journal.

## Competing interests

The authors declare that they have no competing interests.

## Authors' contributions

QML and JHK summarized the case notes, performed the literature search and were major contributors in writing the manuscript. All authors read and approved the final manuscript.
